# G6PD deficiency among malaria-infected national groups at the western part of Myanmar with implications for primaquine use in malaria elimination

**DOI:** 10.1186/s41182-021-00339-7

**Published:** 2021-06-09

**Authors:** Kay Thwe Han, Zay Yar Han, Kyin Hla Aye, Khin Thet Wai, Aung Thi, Liwang Cui, Jetsumon Sattabongkot

**Affiliations:** 1 Parasitology Research Division, Department of Medical Research (DMR), No. 5 Ziwaka Road, Yangon, 11191 Myanmar; 2 DMR, No. 5 Ziwaka Road, Yangon, 11191 Myanmar; 3 National Malaria Control Program, Department of Public Health (DoPH), Naypyitaw, Myanmar; 4 Department of Internal Medicine, University of South Florida, Tampa, USA; 5 Mahidol Vivax Research Unit (MVRU), Faculty of Tropical Medicine, Mahidol University, Bangkok, Thailand

## Abstract

**Background:**

Glucose 6-phosphate dehydrogenase deficiency (G6PDd) plays a central role in readiness assessment for malaria elimination in Myanmar by 2030 that includes primaquine (PQ) use. The risk of hemolysis in G6PDd individuals hampers the widespread use of primaquine safely in malaria-infected patients. In the pre-elimination era, it is important to screen initially for asymptomatic malaria in combination with G6PD deficiency by applying more sensitive diagnostic tools. Therefore, this study examined the proportion of G6PDd and the distribution of G6PD genotypes among malaria-infected national groups in Myanmar before initiation of malaria elimination strategies.

**Methods:**

A cross-sectional study in one township each with high malaria burden from two states in the western part of Myanmar, was conducted during 2016-2018, and 320 participants (164 Rakhine and 156 Chin National groups) were recruited. We used RDT and ultrasensitive polymerase chain reaction (us PCR) method to confirm malaria infection, and a G6PD RDT(CareStart) to detect G6PDd and PCR/restriction fragment length polymorphism (RFLP) method to confirm the variant of G6PDd for genotyping. G6PD enzyme activity was measured by G6PD Biosensor (CareStart).

**Results:**

Malaria positivity rates detected by RDT were lower than those detected by us PCR in the combined samples [13% (42/320) vs. 21% (67/320)] as well as in the Rakhine samples [17% (28/164) vs. 25% (41/164)] and in Chin samples [9% (14/156) vs. 17% (26/156)]. G6PD deficiency rates were approximately 10% in both the combined samples and specific national groups. For G6PD enzyme activity in the combined samples, G6PDd (defined as < 30% of adjusted male median) was 10% (31/320) and severe G6PDd (< 10% of AMM) was 3% (9/320). Among malaria-infected patients with positive by both RDT and usPCR, G6PDd was less than 20% in each national group. G6PD genotyping showed that the G6PD Mahidol (G487A) was the major variant.

**Conclusions:**

The varying degree of G6PDd detected among malaria-infected national groups by advanced diagnostic tools, strongly support the recommend G6PD testing by the National Malaria Control Program and the subsequent safe treatment of *P. vivax* by primaquine for radical cure. Establishing a field monitoring system to achieve timely malaria elimination is mandatory to observe the safety of patients after PQ treatment.

## Background

Malaria elimination is the interruption of local transmission to the zero incidence of indigenous cases of a specified malaria parasite species in a defined geographic area. Continued measures are required to prevent re-establishment of transmission as updated by the World Health Organization (WHO) in 2018 [1]. Malaria Policy Advisory Committee (MPAC) of WHO has considered malaria elimination as technically and operationally feasible at a reasonable cost, and had recommended adopting a goal of malaria elimination in the Greater Mekong Subregion (GMS) by 2030 [2]. Malaria elimination is recognized as requiring a multi-pronged approach. In the GMS, there has been a substantial reduction in the incidence of malaria over the past decade. The changing epidemiological context indicates low endemicity and predominance of asymptomatic and submicroscopic infections that require advanced diagnostic tools to accurately underscore the problem. In addition, the prevalence of *Plasmodium vivax* is increasing relative to *Plasmodium falciparum* [3–5].

As a member of the GMS, Myanmar plans to eliminate malaria by 2030 in accordance with the regional elimination goal. As of 2017, 291 out of 330 townships in the country are malaria-endemic, with 44 million population at risk of malaria. The National Malaria Control Program (NMCP) reported 85,019 cases and 30 deaths in 2017 [6]*.* Malaria is particularly prevalent in the border and hard to reach areas of Myanmar where certain national groups reside. While *P. falciparum* rate has been declining, *P. vivax* is emerging and dominating in some areas. Although the therapeutic efficacy of currently used artemisinin combination therapy (ACT) was well retained in Buthidaung [7], Buthidaung was still the major contributor of malaria burden in Rakhine State in 2017 with the annual parasitic incidence (API) ranking of third. In that report, the API in Chin State was the highest with around 33 [6]. Chemotherapeutic agents play a pivotal role to cut disease transmission by killing the sexual stage of malaria parasites. Primaquine (PQ), an 8-aminoquinoline compound, is the only drug currently available active against all stages of *P. falciparum* gametocytes as well as *P. vivax* hypnozoites [8].

According to the National Malaria Treatment Guideline released in 2017 [9], 0.75 mg/kg single dose of PQ is prescribed with the first dose of artemisinin combination therapy (ACT) for *P. falciparum* malaria and 0.25 mg/kg/day for 14 days for all non-*P. falciparum* malaria. However, a growing body of evidence indicates that malaria control program personnel should provide PQ with caution especially in region with known high glucose 6-phosphate dehydrogenase deficiency (G6PDd) rates which would require mandatory testing for G6PPD before primaquine treatment [10].

G6PDd is an inherited, X-linked recessive trait caused by single nucleotide polymorphisms (SNPs) that impair the function of the G6PD enzyme. The risk of hemolysis in G6PDd hampers the widespread use of PQ safely for malaria elimination. The deficiency remains silent until exposure to certain infections, food, chemicals, or drugs which can cause life-threatening acute hemolytic anemia (AHA). The severity of AHA can range from self-limiting to life threatening depending on the degree of impairment of G6PD enzyme activity [11, 12].

Malaria elimination is achievable through focused efforts to address the asymptomatic and chronic infection reservoirs of *P*. *falciparum* and *P. vivax* [13]. Asymptomatic malaria infections are generally accepted as malarial parasitemia of any density, in the absence of fever or other acute symptoms in individuals who have not received recent antimalarial treatment [14]. Asymptomatic malaria cases, primarily low parasitemic are undetectable by conventional RDT and pose an immense challenge in the GMS for elimination as the reservoir of malaria infection leading to the persistence of malaria transmission [3, 4].

In pre-elimination era, it is crucial to explore the burden of asymptomatic malaria by applying more sensitive diagnostic tools than at present. As such, there is a development of advanced diagnostic molecular methods to detect low density parasitemic malaria [15, 16]. The ultrasensitive (us) RT-PCR method is an advanced molecular tool about 10,000-fold lower detection limit than RDTs to detect low parasite density malaria [15]. This highly sensitive PCR technique reported an unexpectedly high prevalence of asymptomatic malaria in South East Asia [17].

Whatsoever, the success of malaria elimination will rely on the existing disease burden assessment and the readiness of the health care delivery system and the disease control program [18]. In this connection, the national groups in Myanmar have the likelihood of different rates of human genetic enzymopathies like G6PDd. The earlier studies in Myanmar reported G6PDd in five out of eight national groups (Mon, Bamar, Kachin, Kayin, and Shan) in malaria-endemic areas, and diagnostic methods used were variable [19–21]. More specific detection is essential by applying advanced techniques in recent years for asymptomatic malaria and G6PD deficiency rates. In upper Myanmar, Lee et al. [22] elucidated the G6PDd of 20% in 252 samples followed by the ascertainment of G6PD variants. But the study did not include an accurate identification of G6PD enzyme measurement. More specific detection among samples from GMS countries is applied using molecular laboratory techniques in recent years [23]. In the malaria elimination era, accurate measures of G6PD deficiency (G6PDd), and genetic variants remain unexplored in different geographical settings with varying degree of drug resistance and a relatively higher malaria burden in western border of Myanmar. The study in Chittagong hill tracts in proximity to the western border of Myanmar recently reported the wide range of G6PD activities among ethnic groups that justified routine G6PDd testing to guide 8-aminoquinoline based radical (primaquine) in comparable settings [24]. Among others, there is an urgency for the readiness of the NMCP in Myanmar to make evidence-based decisions for malaria elimination by adding measures to detect G6PDd explicitly in the national groups along the western border. Therefore, this study examined the proportion of G6PD deficiency and the distribution of G6PD genotypes among malaria-infected Rakhine and Chin national groups across the western part of Myanmar.

## Methodology

### Study design and duration

**Fig. 1:**
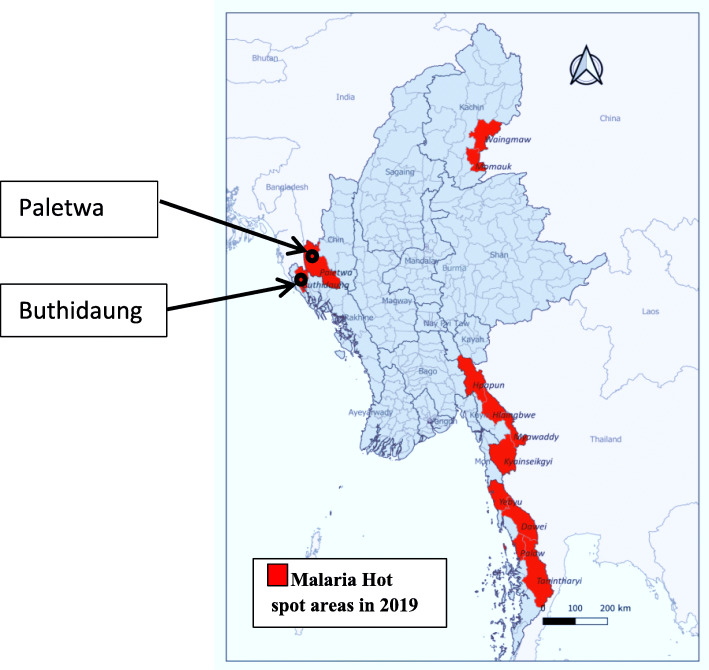
Map showing study sites among malaria hot spot areas of Myanmar in 2019

A cross-sectional study with prospective data collection was carried out in Buthidaung township, Rakhine State, and Paletwa township, Chin State, between 2016 and 2018 (Fig. 1).

### Study setting and study population

There are 14 states/regions in Myanmar and a union territory where the national groups concentrate mostly in Kachin, Kayah, Kayin, Chin, Rakhine, Mon, and Shan States [25]. According to the VBDC annual report 2018 [26], Rakhine State had the highest malaria burden that contributed a quarter of the total malaria cases of the country, whereas Chin State had the third-highest burden accounted for 16%. Buthidaung township in Rakhine State is located at latitude 21° 02′ N (north) and longitude 92° 35′ E (east) in proximity to the Myanmar-Bangladesh border. There were 1798 malaria cases reported during 2018 (up to November). It has a high malaria burden particularly for some villages occupied by Rakhine national groups [26]. Paletwa township is located at latitude 28° 18′ N (north) and longitude 92° 51′ E (east), closed to the Myanmar-India border (see map) (Fig. 1). This township was purposely selected for its high malaria prevalence and most of the residents were Chin national groups [26]. According to the 2014 National Census [25], the total population was 55,545 in Buthidaung and 64,791 in Paletwa. The study population for this research comprised two national groups; Rakhine and Chin resided in the study sites.

### Sample size calculation and the sampling procedure

**Fig. 2:**
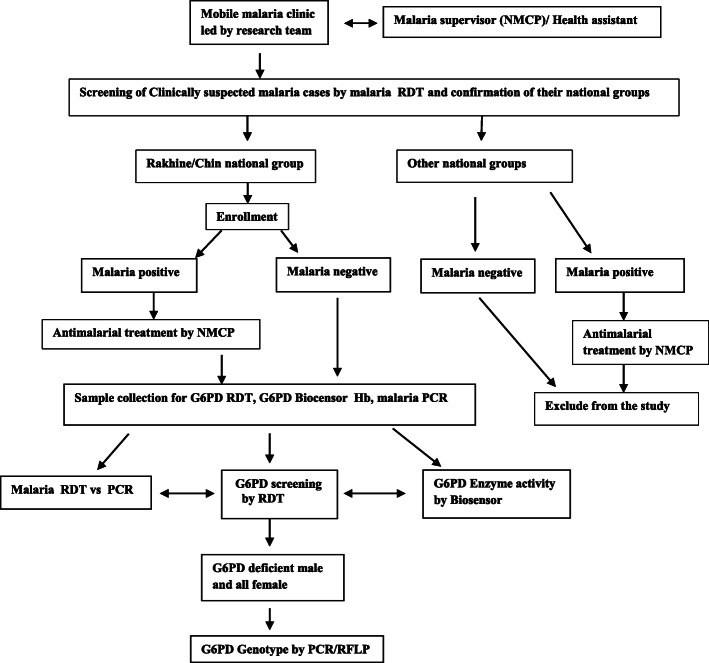
Participant recruitment and study procedure

Of 2958 and 22,421 slides examined by the NMCP in Chin and Rakhine States, the annual slide positivity rates were 16.87% and 17.76% respectively [27]. In line with Jalloh et al, in 2004 [28], basing upon the assumption of the prevalence of G6PD deficiency ranged between 4 and 25% in Myanmar and 20% among the Rakhine national group in Sittwe, Rakhine State, the G6PD deficiency prevalence in malaria (pi 1) is 10 to 25% and the non-malaria was less by half (pi 2) = 5 to 12.5%. For two sided study population at the significant level (∝) = 0.05, group1 population (π1) = 0.25, group 2 population (π2) = 0.125, odd ratio = 0.0429, setting the power of study at 80%, the expected sample size included 168 for malaria-infected, and the equal number for non-malaria patients. Eventually, the research team recruited a feasible sample of 320 participants (164 Rakhine and 156 Chin national groups) attending the mobile malaria clinic in each study site. Each mobile team comprised one research officer, one malaria inspector and two field technicians. The teams screened for malaria by RDT among the clinic attendees and verified the nationality being witnessed by the village elders/village administrative authorities. Clinic attendees within the age range of 2-56 years, of either Rakhine or Chin national were enrolled after obtaining informed consent. The attendees of other national groups were treated according to their malaria RDT results and were excluded from the study (Fig. 2).

### Diagnostic methods

The flow diagram of diagnostic methods used was depicted in Fig. 2.

Three to five drops of capillary blood sample were collected aseptically via finger prick from the participants to screen malaria infection, G6PD deficiency, and measure G6PD enzyme activity and hemoglobin level on site by trained technicians. Further, three to five drops of blood were collected onto 3MM filter paper for molecular diagnosis of malaria and genotyping of G6PD deficiency in the Department of Medical Research (DMR), Yangon, Myanmar.

#### Malaria infection diagnosis

Malaria infection was screened by using RDT (*SD Pf/Pv*) and confirmed by ultrasensitive PCR method described by Adam et al. [15] to detect very low density malaria parasites. The nucleic acid from collected dried blood spot was extracted by using the new extraction method described by Zainabadi [29]. A highly sensitive method for detecting *P. falciparum* and *P. vivax* and 18S ribosomal RNA from DBS was developed by empirically optimizing nucleic acid extraction conditions. The limit of detection (LoD) was less than 16 parasites/mL for *P. falciparum* and 19.7 copies/μL for *P. vivax* (using a plasmid surrogate), about 10,000-fold lower than RDTs [29].

#### G6PD deficiency detection

The RDT test (CareStart G6PD RDT) has been applied to screen G6PD deficiency. It is a chromatographic test to detect G6PD enzyme deficiency qualitatively. The underlying technology of RDT is based on the reduction of nitro blue tetrazolium (colorless) to formazan (dark color), and the pink color is produced if the subject has normal G6PD and no color is produced if the subject is G6PD deficient. The test procedure in brief includes adding 2 μl of capillary blood into the sample well and then adding two drops of buffer into the buffer well of the device. The result is ready after 10 min. The pink colored test is taken as G6PD normal, no color or very faint color is G6PD deficient, and remaining blood in the window shows the invalidity of the test. CareStart G6PD RDT has been validated among healthy volunteers in Myanmar during 2015-2016. It was reported that CareStart RDT showed sensitivity over 95% and specificity over 90% compared to fluorescent spot test (FST) [30].

#### G6PD enzyme activity measurement

G6PD enzyme activity was measured by CareStart G6PD Biosensor, which was undertaken on capillary blood in the field. For each test, a single use strip was applied to record the displayed result. Sensitivity and specificity for detecting G6PD activity < 30% was reported as 0.19 (95% CI, 0.12–0.29) and 0.99 (95% CI, 0.98–0.99) for Biosensor [31] and as 100% and 92% for 30% G6PD activity [32]. Enzyme activity was presented as IU per existing gram hemoglobin.

#### Hemoglobin level determination

HemoCue™ photometer (Hb201+, Angelholm, Sweden) was used to measure the hemoglobin level.

#### G6PD genotyping

Dried blood spot samples of G6PD deficient patients determined by G6PD RDT, and all female samples were subjected to genotypic analysis applying the method described by Laosombat et al. 2005 [33]. DNA was extracted from G6PD deficient samples by chelex method. Extracted DNA samples were genotyped applying the specific set of primers, PCR programs, and restriction enzymes. Ten common G6PD variants in South East Asia [34], namely, G6PD Gaohe A95G, G6PD Chinese 4 G392T, G6PD Mahidol G487A, G6PD Chinese 5 C1024T, G6PD Coimbra C592T, G6PD Viangchan G781A, G6PD Union C1360T, G6PD Canton G1376T, G6PD Seattle G844C, and G6PD Kaiping G1833A were genotyped.

### Data entry and analysis

The survey data were double entered in EPI Data entry software (version 3.1, Epidata Association, Odense-Denmark) for validity and analyzed by SPSS (version 22.0, IBM Corporation, New York, USA). Descriptive statistics were used to examine the demographic characteristics of the study population and the observed overall prevalence ratio and 95% confidence intervals (CI) of malaria infection and G6PD deficiency. Further cross-tabulations were done, and the chi-square test was used to compare gender, malaria infection, and G6PD deficiency. *P* ≤ 0.05 was considered significant. The adjusted male median (AMM) was calculated and defined as 100% G6PD activity for the entire study population [11]. There were three categories of G6PDd as demarcated by AMM value: individuals were categorized as being G6PD *deficient* if their enzyme activity was less than 30% of the AMM and rated as severe if the AMM was < 10%; G6PD intermediate if enzyme activity was between 30 and 80% activity, and G6PD normal if enzyme activity exceeded 80%.

### Ethical considerations

The Ethics Review Committee, Department of Medical Research, Ministry of Health and Sports, Myanmar, approved this study. The written informed consent was obtained before any study procedure. Privacy, anonymity, and confidentiality issues were observed.

## Results

### Background characteristics of the study population

Of 320 participants in the study, Rakhine national group contributed 51% (164/320), and the remaining was Chin national group. In the combined sample, the proportion of male was 52% (165/320), and the median age was 22.5 years (IQR 12-39) in combined samples, and 21 years (IQR 12-40.75) and 23 years (IQR 13-37) in Rakhine and Chin respectively.

### Prevalence of malaria infection

Table 1 compared the prevalence of malaria infection by two methods (RDT and ultrasensitive PCR). As might be expected, us PCR was more likely to detect malaria infection in both national groups compared to RDT (detecting malaria infection in 25 of 275 RDT-negative samples).

**Table 1:** Malaria positivity rate determined by RDT in two national groups, Myanmar (2016-2018)

Test	Rakhine		Chin		Combined sample	
	RDT (*n* = 164)	usPCR (*n* = 164)	RDT (*n* = 156)	usPCR (*n* = 156)	RDT (*n* = 320)	usPCR (*n* = 320)
Malaria positive	28 (17%)	41 (25%)	14 (9%)	26 (17%)	42 (13%)	67 (21%)
Specific species						
*Plasmodium falciparum* (*Pf*)	24 (15%)	31 (19%)	6 (4%)	13 (8%)	30 (9%)	44 (14%)
*Plasmodium vivax* (*Pv*)	3 (2%)	5 (3%)	7 (4%)	11 (7%)	10 (3%)	16 (5%)
Mixed (*Pf and Pv*)	1 (0.6%)	5 (3%)	1 (0.6%)	2 (1%)	2 (0.6%)	7 (2%)

Malaria positivity rates detected by RDT were more likely to be lower than those detected by us PCR in the combined febrile samples [13% (42/320) vs. 21% (67/320)] as well as in the specific samples of Rakhine national group [17% (28/164) vs. 25% (41/164)] and Chin national group [9% (14/156) vs.17% (26/156)]. Regarding species prevalence, *P. falciparum* dominated in the Rakhine national group, whereas *P. vivax* equalized the *P. falciparum* in the Chin national group.

### G6PD deficiency rate determined by CareStart RDT

The G6PD deficiency rate as determined by CareStart RDT was 10% (32/320) in the combined sample, and similar rates were noted for both Rakhine and Chin national groups [9.8% (16/164) vs. 10.3% (16/156)] respectively. In the combined sample, males were two times more likely than females to identify as G6PD deficient [13.3% (22/165) vs. 6.5% (10/155)] and more than two times more likely to be malaria-infected than females (6.87% vs. 3.12%; crude OR = 2.06; 95% CI, 1.01-4.22; *p* = <0.05).

### G6PD enzyme activity determined by CareStart G6PD Biosensor

CareStart G6PD Biosensor ascertained the median value of G6PD enzyme activity (U/gHb) as 6.6 (IQR 5.3-7.9) for the combined samples and as 6.3 (IQR 4.6-7.5) in the male population. The adjusted male median (AMM) value of G6PD enzyme activity was calculated after exclusion of G6PD enzyme activity values of 7 male patients having < 10% of male median value. AMM was 6.4 U/gHb (IQR 5.0-7.6) (Table 2). Applying this AMM value, G6PD deficiency rate (< 30% of AMM) was 9.7% (31/320) and severe G6PD deficiency rate (< 10% of AMM) was 2.8% (9/320) in the combined samples. Intermediate deficiency (30-80%) was detected in 14.1% (45/320) of combined samples.

**Table 2:** G6PD enzyme activity determined by Biosensor among males in the combined sample of two national groups, Myanmar (2016-2018)

G6PD enzyme activity (U/gHb)	Combined sample (*n* = 320)	Male (*n* = 165)	Adjusted male median^a^ (*n* = 158)
Median (U/gHb)	6.6	6.3	6.4
IQR (interquartile range)	5.3-7.9	4.6-7.5	5.0-7.6

^a^Excluded enzyme activity values of 7 male patients having < 10% of male median value

### G6PD deficiency rate among malaria positive patients

According to Table 3, among malaria RDT positive patients, 16.7% (7/42) were G6PD RDT positive (deficient) G6PD RDT and among malaria PCR positive patients, 14.9% (10/67) were G6PD RDT positive.

**Table 3:** Proportion of G6PD deficiency among malaria-infected populations in the combined sample of two national groups, Myanmar (2016-2018)

Malaria status	G6PD RDT negative (G6PD normal)	G6PD RDT positive (G6PD deficiency)	Combined study population
Malaria RDT negative	253	25	278
Malaria RDT positive	35	**7 (16.7%)**^a^	42
Malaria PCR negative	231	22	253
Malaria PCR positive	57	**10 (14.9%)**^b^	67

^a^Pearson chi-square value = 2.38, *p* = 0.122, ^b^Pearson chi-square value = 2.28, *p* = 0.13

### G6PD genotypes and phenotypes

PCR/RFLP method was used to genotype all 22 male samples with G6PD RDT positive and all female samples. Nineteen out of 22 male samples harbored Mahidol variant (G6PD G487A), and 3 samples were wild type.

Among 155 female samples consisted 10 G6PD RDT positive and 145 G6PD RDT negative samples. Overall, 85.8% of female participants harbored wild type genotype (133 out of 155) and mutant G6PD was found in 22 samples (14.2%). Mutant female samples included 10 G6PD RDT positive and 12 G6PD RDT negative. G6PD RDT positive 10 samples showed homozygous mutation of Mahidol variant and 9 out of 10 samples had G6PD enzyme activity < 30% of AMM and one sample had 39% of AMM. In the remaining 12 samples, one sample was heterozygous Chinese-4 variant and 11 were heterozygous Mahidol variant. In relation to enzyme activity, 4 out of 12 samples showed intermediate deficiency (30-80% of AMM) and 8 samples had normal G6PD enzyme activity (> 80% of AMM) (Table 4).

**Table 4:** Comparison of G6PD genotypes and phenotypes

Gender	Phenotype (by RDT)		Genotype		
	Normal	Deficient	Wild type	Mahidol	Chinese 4
Female	145		133	11	1
		10	0	10	0
Male	Not done				
		22	3	19	0

## Discussion

There was a significant decrease in symptomatic malaria cases during 2018 in Myanmar due to intensive malaria control efforts incorporating multidispronged approach. In the present study, malaria positivity rates of the Rakhine national group residing at Buthidaung was higher than the Chin national group residing at Paletwa, both by RDT and us PCR. RDTs have long been used to screen clinically suspected malaria cases for decades. In this study, conventional RDT missed 25 cases of low-density parasitemic malaria cases which were further detected by us PCR. Malaria elimination may be possible only with concerted attempts to identify asymptomatic and chronic infection [13]. To explore low-level parasitemia undetectable by conventional RDTs, more sensitive diagnostic tools are applicable to eliminate all malaria reservoirs in line with malaria elimination strategies in the GMS including in Myanmar [3, 4, 8]. In the pre-elimination stage, disease burden assessment is critical for setting up and monitoring the appropriate elimination strategies.

As part of the readiness assessment, both malaria burden and existing G6PD deficiency rates among various national groups residing in high endemic, border areas should be explored before widespread use of transmission-blocking agents like primaquine [8]. The applicability of G6PD RDT was validated on healthy volunteers in Myanmar in 2015 and the reported sensitivity was over 95% and specificity over 90% compared to the fluorescent spot test (FST) [30]. One study that looked into the distribution of G6PD mutation in South East Asia reported that 10.8% of the Shan national group was G6PD deficient [34]. Another study reported higher rates of G6PDd among Shan and Rakhine national groups in malaria endemic areas (15.4% and 20.2% respectively, by the rapid assessment) [28]. A study in the southern part of Myanmar reported a G6DP deficiency rate of 12% in Mon and 10% in Burmese national groups by FST method [19]. In Karen migrant and refugee camp along Myanmar-Thai border area, the overall G6PD deficiency rate determined by FST was 13.7% [20]. A study among the Kachin national group along the Myanmar-China border area in the northern part of Myanmar revealed aG6PD deficiency rate of 29.3% as determined by FST in 2013 [21]. Another study conducted on a Skaw Karen population reported 28% of males asG6PD deficient which was confirmed by FST method [35]. Subsequently, in upper Myanmar, the rate of G6PDd among malaria patients was reported as 19.9% [22]. Among the GMS countries, the reported prevalence of G6PDd in 2013 was the highest in Cambodia (18.8%), followed by Myanmar (15.8%) with rates of less than 10% in Vietnam (8.9%), Lao PDR (8.1%), and Thailand (7.3%) [23]. The earlier studies reported the G6PD deficiency rates of different national groups of Myanmar within 10-20%. The finding of the study was well within that range (9.8% in Rakhine and 10.3% in Chin national groups).

Among those with G6PD deficiency in the combined samples in this study, 68.8% were male (crude OR = 2.06; 95% CI, 1.011-4.222; *p* = <0.05) which was consistent with the Thailand study [36]. Applying G6PD Biosensor, enzyme activity was also determined based on hemoglobin (gHb) in the present study. The application of G6PD RDT in malaria patients allows for identification of vulnerable population with hemolytic potential, defined as less than 30% of enzyme activity, before primaquine treatment. The proportion of the population with < 30% of enzyme activity was 9.7% in this study. The difference in prevalence rates as determined by the two different methods (G6PD RDT vs*.* Biosensor) was negligible (9.7% vs. 10%). The performance of CareStart RDT is satisfactory to identify individuals with < 30% of G6PD enzyme activities with high negative predictive value (0.99, 95% CI, 0.94-1.00), suggesting its applicability to guide PQ treatment [37].

G6PD variants identified by PCR/RFLP revealed that all of the G6PD deficient samples by RDT turned out to be Mahidol variants. This specific variant was frequently reported as the more common variant, especially among different national groups of Myanmar. G6PD mutation study performed in Myanmar reported the Mahidol variant as the most common variant (90% of the affected population) [38] which was similar to the study conducted among Skaw Karen group [35]. Another two studies conducted on G6PD variants in Myanmar reported that more than 90% of G6PD deficient subjects were Mahidol variants [20, 39]. Among the G6PD-deficient Mon population, 12 out of 19 were found to be Mahidol variant, and one each of G6PD Kaiping variant and G6PD Mediterranean were also identified [19]. Also, in upper Myanmar, Lee et al. in 2018 [22] reported that the Mahidol variant was the most common variant. In the present study, ten common G6PD variants were genotyped but the Mahidol variant was found specifically in the G6PD deficient samples.

Among the female samples, mutation was detected in 22 samples (14.2%) which were comprised of 21 G6PD Mahidol and one Chinese-4 variant. All G6PD RDT positive female samples (n = 10) harbored homozygous Mahidol type. In relational to enzyme activity measured by Bisensor, 9 out of 10 samples had G6PD enzyme activity < 30% of AMM and one sample showed enzyme activity 39% of AMM. G6PD enzyme activity is presented as IU per gram of existing hemoglobin. Very low level of Hb may result in higher enzyme activity than actual. This may be the reason for the discrepancy in G6PDd rates between Biosensor and other diagnostic methods. Future studies with a larger sample size could explore the distribution of other G6PD variants to explore novel mutations among population. As WHO recommends for G6PD testing for the safe use of primaquine [40], G6PD deficiency rates among malaria-infected population is of interest in order to determine whether G6PD screening is required for primaquine use. The proportion of G6PDd among malaria patients (16.7% by RDT, 14.9% usPCR) in this study in two national groups of the western part of Myanmar indicated G6PD testing or special interventions and/or alternative strategies may be required to achieve malaria elimination goal in Myanmar.

### Strengths and limitations

This is the first study in Myanmar exploring the prevalence of G6PD deficiency with exact measurements using advanced molecular tools among two national groups in the western part of the country with high malaria burden to inform NMCP for its applicability in moving toward malaria elimination. The FST Gold standard testing for G6PD deficiency was unable to be applied in this study as the facility was limited in the study areas.

## Conclusions

The varying degree of G6PD deficiency among malaria-infected national groups supports the strongly recommend G6PD testing by the National Malaria Control Program and the subsequent safe treatment of *P. vivax* for radical cure. Establishing the field monitoring system to achieve timely malaria elimination is mandatory to observe the safety of patients after PQ treatment.

## Data Availability

The data set supporting the relevant findings and conclusions of the study is available upon reasonable request.

## Abbreviations

G6PD: Glucose 6 phosphate dehydrogenase
NMCP: National Malaria Control Program
WHO: World Health Organization
RDT: Rapid diagnostic test
usPCR: Ultrasensitive polymerase chain reaction
PCR/RFLP: Polymerase chain reaction (restriction fragment length polymorphism)
FST: Fluorescent spot test
AMM: Adjusted male median
GMS: Greater Mekong Subregion
