# Correction: G6PD deficiency among malaria-infected national groups at the western part of Myanmar with implications for primaquine use in malaria elimination

**DOI:** 10.1186/s41182-023-00498-9

**Published:** 2023-02-06

**Authors:** Kay Thwe Han, Zay Yar Han, Kyin Hla Aye, Khin Thet Wai, Aung Thi, Liwang Cui, Jetsumon Sattabongkot

**Affiliations:** 1 Parasitology Research Division, Department of Medical Research (DMR), No. 5 Ziwaka Road, Yangon, 11191 Myanmar; 2 DMR, No. 5 Ziwaka Road, Yangon, 11191 Myanmar; 3 Department of Public Health (DoPH), National Malaria Control Program, Naypyitaw, Myanmar; 4 Department of Internal Medicine, University of South Florida, Tampa, USA; 5 Mahidol Vivax Research Unit (MVRU), Faculty of Tropical Medicine, Mahidol University, Bangkok, Thailand

**Correction to: Tropical Medicine and Health (2021) 49:47** **https://doi.org/10.1186/s41182-021-00339-7**

Following publication of the original article [1], the authors reported that the author affiliations need to be updated.

The original author affiliations were:

^1^ Parasitology Researsch Division, Department of Medical Research (DMR), No. 5 Ziwaka Road, Yangon 11191, Myanmar.

^2^ DMR, No. 5 Ziwaka Road, Yangon 11191, Myanmar.

^3^ National Malaria Control Program, Department of Public Health (DoPH), Naypyitaw, Myanmar.

^4^ Department of Internal Medicine, University of South Florida, Tampa, USA.

^5^ Mahidol Vivax Research Unit (MVRU), Faculty of Tropical Medicine, Mahidol University, Bangkok, Thailand.

The correct author affiliations have been updated in this Correction.

The original article [1] has been corrected.
